# Association between serum uric acid and depressive symptoms stratified by low-grade inflammation status

**DOI:** 10.1038/s41598-021-99312-x

**Published:** 2021-10-14

**Authors:** Sang Jin Rhee, Hyunju Lee, Yong Min Ahn

**Affiliations:** 1 Department of Neuropsychiatry, Seoul National University Hospital, 28 Yongon-Dong, Chongno-gu, Seoul 03080 Republic of Korea; 2 Department of Psychiatry, Seoul National University College of Medicine, 28 Yongon-Dong, Chongno-gu, Seoul 03080 Republic of Korea; 3 Institute of Human Behavioral Medicine, Seoul National University Medical Research Center, 28 Yongon-Dong, Chongno-gu, Seoul 03080 Republic of Korea

**Keywords:** Depression, Diagnostic markers

## Abstract

Despite increasing evidence for an association between circulating uric acid (UA) and depression, the directionality of this association remains unclear and is potentially moderated by low-grade inflammation. Thus, the present study aimed to investigate the cross-sectional association between serum UA concentration and depressive symptoms in Korean individuals with and without low-grade inflammation, as measured using serum high-specific C-reactive protein (hs-CRP) levels. The final study sample comprised 4188 participants, aged 19–79 years, from the Korea National Health and Nutrition Examination Study 2016. Data on serum uric acid (UA) concentrations, serum hs-CRP levels, Patient Health Questionnaire-9 (PHQ-9) scores, and relative covariates were retrieved. Negative binomial regression with adjustment for the complex sample design was used to analyze the associations. After adjusting for covariates, log-transformed serum UA concentrations and total PHQ-9 scores were positively associated (incidence rate ratio [IRR] = 1.34 [95% confidence interval [CI] = 1.09–1.66]) for participants without low-grade inflammation and inversely associated (IRR = 0.64 [95% CI = 0.45–0.92]) for participants with low-grade inflammation. In conclusion, the direction of the association between serum UA and depressive symptoms was the opposite in participants with and without low-grade inflammation. The study has the limitation of potential uncontrolled confounders.

## Introduction

Uric acid (UA), the end product of purine metabolism, is primarily known for its association with gout^1^. However, there is increasing evidence of its association with the central nervous system, including depression^2^. Most previous studies investigating the association between UA and depression/depressive symptoms reported an inverse association^3–6^. However, a recent report on adolescents found that UA levels positively correlated with depression^7^. UA may have neuroprotective effects, as it is the major antioxidant in human blood^8^. UA also functions as an intracellular pro-oxidant^9^, and high serum UA levels are a well-known risk factor for cardiovascular risks^10, 11^; these are also associated with depression^12^. The dual anti- and pro-oxidant properties of UA imply that the association between UA and depression may be moderated by tertiary factors.

Inflammation is also known for its association with psychiatric diseases^13^. Especially low-grade inflammation has been thoroughly studied as a potential mechanism of mood disorders, including depression^14, 15^. However, these studies tend to exclude participants with high-level inflammatory states like acute infection, since the symptoms of the acute condition itself can manifest like depression. The role and importance of inflammation in these disorders have been investigated, and recent studies have shown that low-grade inflammation and cardio-metabolic disturbances are associated with mood symptoms^16, 17^.

Recently, numerous studies have focused on the interaction between UA and inflammation in certain conditions, including hypertension^19^, albuminuria in type 2 diabetes^20^, and even mortality^21^. For instance, low-grade inflammation was shown to significantly amplify the effect of UA on albuminuria in type 2 diabetes, moderating the association^20^. As UA has potential interactions with low-grade inflammation, its influence on depression might also be moderated by low-grade inflammation, which would further explain previous discrepancies. UA’s anti-and pro-oxidant properties in depression might depend on the level of low-grade inflammation.

Given this background, the present study sought to clarify the cross-sectional association between UA and depressive symptoms in the Korean general population. We hypothesized that the association between UA and depressive symptoms would differ between individuals with and without low-grade chronic inflammation, as measured using serum high-specific C-reactive protein (hs-CRP) levels.

## Results

### Demographic and clinical characteristics by low-grade inflammation state (Table 1)

**Table 1:** Demographic and clinical characteristics of the study subjects stratified by low-grade inflammation status.

	Without low-grade inflammation^a^ (hs-CRP < 1.0 g/ml) (n = 3129, Size = 26,072,526)	With low-grade inflammation^a^ (hs-CRP ≥ 1.0 g/ml) (n = 1059, Size = 8,333,353)	Statistics^a,b^	df	*P* value^a,c^
Age, mean [95% CI], years	44.99 [44.14–45.83]	47.67 [46.40–48.93]	t = 4.36	165	< 0.001***
Sex			F = 11.65	1	0.001**
Women, *n* (%)	1868 (50.5%)	558 (43.4%)			
Region			F = 0.89	1	0.35
Rural, *n* (%)	572 (13.4%)	232 (14.9%)			
Income			F = 10.14	1	0.002**
Less than the 1st quartile of housing income, *n* (%)	463 (12.8%)	228 (17.1%)			
Education			F = 1.89	1	0.17
At least a college graduate, *n* (%)	1242 (42.9%)	355 (40.3%)			
Unemployment, *n* (%)	1160 (34.1%)	433 (35.8%)	F = 0.59	1	0.44
Marital status			F = 1.64	1.92	0.20
Currently married, *n* (%)	2271 (66.7%)	772 (68.2%)			
Previously married, *n* (%)	322 (8.2%)	139 (9.7%)			
Never married, *n* (%)	536 (25.1%)	148 (22.1%)			
Alcohol use,* n* (%)	1778 (62.6%)	551 (57.6%)	F = 6.11	1	0.014*
Current smoker, *n* (%)	488 (20.1%)	212 (26.4%)	F = 11.22	1	0.001**
No aerobic exercise, *n* (%)	1620 (48.1%)	609 (52.8%)	F = 5.15	1	0.025*
History of cancer, *n* (%)	143 (3.6%)	51 (3.9%)	F = 0.19	1	0.67
History of arthritis, *n* (%)	230 (5.1%)	102 (6.8%)	F = 4.14	1	0.043*
Hypertension, *n* (%)	860 (23.6%)	426 (35.8%)	F = 44.98	1	< 0.001***
Diabetes, *n* (%)	314 (7.8%)	187 (13.8%)	F = 31.85	1	< 0.001***
Hypercholesteremia, *n* (%)	638 (18.0%)	256 (21.0%)	F = 3.97	1	0.048*
Obesity, *n* (%)	928 (29.9%)	563 (54.3%)	F = 111.52	1	< 0.001***
Anemia, *n* (%)	295 (7.9%)	85 (6.2%)	F = 3.20	1	0.08
AST^d^, mean [95% CI], IU/L	20.23 [19.97–20.49]	22.81 [22.12–23.51]	t = 7.61	165	< 0.001***
Creatinine^d^, mean [95% CI], mg/dL	0.814 [0.806–0.822]	0.839 [0.824–0.853]	t = 3.34	165	0.001**
hs-CRP^d^, mean [95% CI], mg/L	0.434 [0.423–0.446]	2.17 [2.08–2.26]	t = 66.71	165	< 0.001***
Daily energy intake^d^, mean [95% CI], kcal	1894 [1854–1935]	1940 [1869–2013]	t = 1.11	165	0.27
Daily protein intake^d^, mean [95% CI], g	65.36 [63.57–67.20]	65.49 [62.87–68.22]	t = 0.09	165	0.93
Uric acid^d^, mean [95% CI], mg/dL	4.82 [4.76–4.88]	5.21 [5.11–5.30]	t = 6.35	165	< 0.001***
PHQ-9 total score, median [interquartile range]	1 [0–3]	1 [0–3]	t = 0.73	164	0.47

hs-CRP = high-sensitive C-reactive protein, df = degree of freedom, CI = confidence interval, AST = aspartate aminotransferase, PHQ-9 = Patient Health Questionnaire-9.

^a^Statistics adjusted for complex sample design.

^b^Categorical variables based on the adjusted-F by Pearson's chi-square tests, continuous variables except the PHQ-9 scores based on the *t* tests, and the PHQ-9 scores based on the design-based Kruskal Wallis tests.

^c^*p* values are significant at α = 0.05 (*), 0.01 (**), and 0.001(***).

^d^Due to the skewed distribution, logarithmic transformation was performed for the statistics, and the CIs were back-transformed.

When compared with those without low-grade inflammation, participants with low-grade inflammation were older, more often current smokers, more often had a history of arthritis, and were less often women, had less household income, less often alcohol users, and less often engaged in aerobic exercise. Additionally, hypertension, diabetes, hypercholesteremia, and obesity were more common in those with low-grade inflammation. When comparing laboratory values, serum aspartate aminotransferase (AST), creatinine, and UA levels were higher in those with low-grade inflammation. Finally, there was no significant difference in total Patient Health Questionnaire-9 (PHQ-9) scores between participants with and without low-grade inflammation.

### Association between serum uric acid concentrations and depressive symptoms by low-grade inflammation state (Table 2)

**Table 2:** The association between uric acid concentrations and depressive symptoms stratified by low-grade inflammation status.

	Without low-grade inflammation (hs-CRP < 1.0 g/ml)					With low-grade inflammation (hs-CRP ≥ 1.0 g/ml)				
	(n = 3129, size = 26,072,526)					(n = 1059, Size = 8,333,353)				
	Incidence Rate Ratio (95% CI)	Design df	*p* value^a^	Alpha	Dispersion^b^	Incidence Rate Ratio (95% CI)	Design df	*p *value^a^	Alpha	Dispersion^b^
PHQ-9 total score^c^	1.34 (1.09–1.66)	165	0.007**	1.36	1.07	0.64 (0.45–0.92)	165	0.016*	1.30	1.09

Analysis done by negative binomial regression with adjustment for complex sample designs.

hs-CRP = high sensitive C-reactive protein, CI = confidence interval, df = degree of freedom, PHQ-9 = Patient Health Questionnaire-9.

^a^*p* values are significant at α = 0.05 (*), 0.01 (**), and 0.001(***).

^b^Dispersion based on Pearson.

^c^Adjusted for age, sex, region, income, education, unemployment, marital status, alcohol use, smoking behavior, aerobic exercise, history of cancer, history of arthritis, hypertension, diabetes, hypercholesteremia, obesity, anemia, serum AST, serum creatinine, serum hs-CRP, daily energy intake, and daily protein intake.

The interaction term between log-transformed serum UA concentrations and the presence of low-grade inflammation was marginally associated with total PHQ-9 scores (*p* = 0.10). Among participants without low-grade inflammation, there was a positive association between log-transformed serum UA concentrations and total PHQ-9 scores (incidence rate ratio [IRR] = 1.34 [95% confidence interval [CI] = 1.09–1.66], df [degree of freedom] = 165, *p* = 0.007), after controlling for previous covariates. However, in those with low-grade inflammation, there was an inverse association between log-transformed serum UA concentrations and total PHQ-9 scores (IRR = 0.64 [95% CI = 0.45–0.92], df = 165, *p* = 0.016).

## Discussion

In the present study, the association between serum UA levels and depressive symptoms was reversed by the presence of low-grade inflammation. In participants without low-grade inflammation, higher UA levels were associated with depressive symptom severity, while in participants with low-grade inflammation, it was lower UA levels which were associated with depressive symptom severity.

Most previous studies reported an inverse association between UA and depression/depressive symptoms^3–6^. One prior study of young participants demonstrated a positive association^7^. This is of particular interest, as those without low-grade inflammation were younger in the present study. Discrepancies between previous studies may be driven by the various study populations’ inflammatory statuses. A previous study from China, which reported an inverse association between UA and depression, was performed in a study population with a mean CRP level higher than that in the low-grade inflammation status group assessed in our study^6^. However, another study from Denmark, which reported an inverse association between UA and depression, was conducted in a population with a median CRP level in-between those with and without low-grade inflammation in the present study^5^, so direct comparison is limited. Notably, the authors suggested that high UA levels may have a paradoxical effect on depression, as depression risk was lower in the third than the fourth (highest) quartile of UA levels in their study^5^.

Additionally, differing results may be due to differing study populations and statistical analysis methods. The present study evaluated depressive symptoms in the general population, while most studies, except the one from China discussed above, enrolled depressed patients. Furthermore, we excluded participants with hs-CRP levels > 10 mg/L. During acute infection, the PHQ-9 may actually reflect acute illness and not depressive symptoms. Finally, unlike previous studies, the present study used a negative binomial regression approach and adjusted for complex sample designs to reflect the distribution of depressive symptom scale scores in the general population. However, direct comparison is limited as previous studies were not stratified by low-grade inflammation.

The opposite direction of the associations between UA and depressive symptoms with and without low-grade inflammation may be due to UA’s dual pro- and antioxidant properties. In participants with low-grade inflammation, UA levels may present antioxidant capacity. Lower antioxidant capacity with inflammation induces oxidative and nitrosative stress, which causes damage to various cellular components including fatty acids, proteins, DNA, and mitochondria^18^. Further, this is known to increase indoleamine dioxygenase, the enzyme that catabolizes tryptophan into neurotoxic kynurenine instead of serotonin^18^, and is also known to cause damage to membrane polysaturated fatty acids and may affect serotoninergic membrane receptors^18^. Finally, this can also induce neurodegeneration, also a known factor of depression^18^. In those without low-grade inflammation, the association between UA and depression was reversed. In this situation, UA levels may present a certain pro-oxidant capacity^9^. The prior study that reported that higher serum UA levels were associated with depression revealed that serotonin levels in the brain were negatively associated with blood UA levels in depressed patients^7^. Pro-oxidant UA may result from increased nucleic acid oxygenolysis in depression^7^. However, the pro-oxidant UA effects also induce inflammation^22^, which CRP may not be able to reflect as the association with oxidative stress differs with inflammatory markers^23^. Recent meta-analyses reported a significant association between inflammation and mood disorders, including depression^14, 15^. Inflammation has also been associated with emotional reactivity in patients even in remission^16^. A recent study revealed that these symptoms are associated with cardiovascular function, glucose metabolism, and lipid metabolism^17^. These cardiovascular disturbances, components of the metabolic syndrome, are all linked to oxidative stress, inflammation, and depressive symptoms^24, 25^. Therefore, further investigation of the complex association between oxidative stress, inflammation, and depressive symptoms is needed^18, 26^.

One additional mechanism that might be considered together with oxidative stress is the purine metabolism pathway. UA is the end product of the purine metabolism; hypoxanthine is catabolized to xanthine, and xanthine to UA by the enzyme xanthine oxidase. A recent study reported an altered purine metabolism in depression and that levels of inosine and guanine, upstream metabolites of xanthine in purine metabolism, were decreased and xanthine levels were increased in patients with depression^27^. The authors suggested that despite the increased xanthine oxidase activity in depression as a compensatory mechanism to oxidative stress, it is insufficient to overcome the UA consumption as an antioxidant, thus leading to decreased UA levels^27^. This compensatory mechanism may in fact be dependent on low-grade inflammation. In people with low-grade inflammation, despite the increase in xanthine oxidase, UA use as an antioxidant may decrease its levels. However, in those without low-grade inflammation, a hyperactive purine metabolism in depression could lead to UA accumulation. Further studies investigating the association between specific purine metabolism pathway metabolites and enzymes based on inflammation status in depression may clarify this issue.

The present study has some limitations. First, it utilized a cross-sectional design such that causality cannot be determined. Despite controlling for diet and exercise, which can influence UA concentrations, other lifestyle factors associated with depression may influence UA levels. Additionally, participants with higher UA have a higher probability of hypertension, and beta-blockers commonly prescribed for hypertension might have a longitudinal association with depression^28^. Longitudinal studies are thus needed to clarify causality. Second, our assessment of depressive symptoms was based on the PHQ-9, which utilizes a self-report measure. However, the PHQ-9 is based on the Diagnostic and Statistical Manual of Mental Disorders and has significant internal consistency and test–retest reliability. Third, there may be other covariates that we did not control. Despite controlling for potential confounders, specific symptoms related to depression such as emotional dysregulation^16^, anxiety^3^, sleep^29^, aggression^30^, and impulsivity^31^ are also known to be associated with circulating UA or hs-CRP levels. As this study was based on data retrieved from the Korea National Health and Nutrition Examination Study (KNHANES), the confounders we could control were limited. Other potential confounders might have influenced the association. Fourth, an even larger sample would enable further stratifications for specific covariates as an additional analysis, which could expand our knowledge between the association of UA and depressive symptoms. However, a larger sample would increase the risk of type I error. Fifth, the interaction term was only marginally significant, even though the stratification was based on a priori hypothesis. Finally, the KNHANES focuses on depression/depressive symptoms, and the influences of other psychiatric disorders were not evaluated.

Despite its limitations, the present study also has its strengths. First, the data presented here reveal an association between serum UA and depressive symptoms dependent on low-grade inflammatory status, which may explain discrepancies with previous studies. Second, we used a negative binomial regression adjusted for a complex sample design using a national, representative population. These analyses maximized statistical power and reflected the distribution of PHQ-9 scores in the general population.

In conclusion, our study revealed an opposite association between serum UA and depressive symptoms in participants with and without low-grade inflammation. This emphasizes the importance of screening for and diagnosing depression in those with serum UA and hs-CRP level imbalances. Further research should use a longitudinal design to clarify the specific mechanisms underlying this relationship and should consider low-grade inflammation as a potential moderator.

## Methods

### Study sample population

Data were retrieved from the KNHANES VII-1 (2016), an annual national representative survey of noninstitutionalized civilians in the Republic of Korea. The KNHANES assesses health and nutritional status and estimates the prevalence of chronic diseases. To this end, the KNHANES uses a complex, multi-stage probability sample design. In total, 192 primary sampling units (PSUs) were selected based on administrative districts and housing types in the Republic of Korea. A systemic sampling system with intra-stratification by age, sex, and residential area was applied to extract 23 households from each PSU. Finally, within each household, those aged ≥ 1 year were eligible to participate.

Data from subjects who participated in the health interviews, health examinations, and the nutritional survey from the KNHANES VII-1 (2016) between January and December 2016 were included. Self-administered and structured questionnaires were used to collect information about participants’ demographic, clinical, and nutritional characteristics, and physical examinations were conducted. The total number of participants in the KNHANES VII-1 was 8150, 6703 of whom participated in all three individual surveys. In our study, only participants 19–79 years of age were included, yielding 4943 participants, as the Patient Health Quesionnaire-9 (PHQ-9) was only conducted for those who were ≥ 19-years old. Excluding participants with missing serum UA and PHQ-9 data yielded 4592 participants, and further excluding those who were pregnant/breastfeeding, those who were being treated for depression as medication is also known to affect UA levels^32^, and those with high-sensitive C-reactive protein (hs-CRP) levels > 10 mg/L indicating an acute infection, yielded 4345 participants. Finally those with missing values for covariates, including those who did not fast ≥ 8 h prior to blood sampling, were excluded. The final sample included 4188 participants.

Ethical approval for survey procedures by the Institutional Review Board of Korea Centers for Disease Control and Prevention was waived as the KNHANES was conducted to improve national government public welfare measures. All methods were carried out in accordance with relevant guidelines and regulations. Written informed consent was obtained from all survey participants. Ethical approval for the study design by the Institutional Review Board of Seoul National University Hospital was waived due to the anonymity and publicly available nature of the data.

### Assessment of serum uric acid concentrations and high-sensitivity C-reactive protein levels

In the KNHANES VII-1, after ≥ 8 h of fasting, blood samples were collected to assess levels of biochemical markers. Venous serum samples were collected in a serum separating tube, centrifuged at 3000 rpm for 15 min, and stored at 2–8 °C. Serum UA and hs-CRP were measured within 24 h of sample collection. Serum UA levels were measured by the colorimetric enzymatic (uricase) method using the Hitachi Automatic Analyzer 7600–210 (Hitachi/Japan)^33^. In four participants, UA levels were below detection limits (< 1.0 mg/dL) and imputed as 0.9 mg/dL. Serum hs-CRP levels were measured by an immunoturbidimetric method using the Cobas analyzer (Roche, Germany). Levels in 40 participants in the final population were < 0.15 mg/L and imputed as 0.149 mg/L. The target monthly coefficient variances for both UA and hs-CRP were set to ≤ 5% and were satisfied. In our study, the presence of low-grade inflammation was defined as hs-CRP level ≥ 1.0 mg/L, which is usually used as a cutoff to distinguish low from intermediate cardiovascular risk^34^. This value was also equal to the highest quartile of hs-CRP levels in the final population.

### Assessment of depressive symptoms

In the KNHANES VII-1, the Korean version of the PHQ-9 was used to assess depressive symptom severity. The PHQ-9 is a nine-item self-report questionnaire. Each question is scored from 0 to 3 points based on the frequency of each symptom. The PHQ-9 has significant internal consistency (α = 0.81–0.86) and test–retest reliability (coefficient = 0.79–0.89)^35, 36^. The primary outcome of the present study was the total PHQ-9 score. It was used as a continuous variable to assess associations with depressive symptom severity and to maintain statistical power.

### Other covariates

Covariates for adjustment were based on previous studies investigating the association between UA and depression/depressive symptoms or known associations with UA or depression/depressive symptoms^3, 5, 6, 37–43^. We controlled for the following health interview covariates; age (continuous variable), sex (men or women), region (rural or urban), income (less than the 1st quartile of housing income or at least the 1st quartile of housing income), education (not a college graduate or at least a college graduate), unemployment (yes or no), marital status (currently married, previously married, not married), alcohol use (yes or no), smoking behavior (current smoker or current non-smoker), aerobic exercise (yes or no), history of cancer (yes or no), and history of arthritis (including osteoarthritis and rheumatoid arthritis; yes or no). Alcohol use was defined as drinking ≥ 1 cup of alcoholic beverage per month for the past year. A current smoker was defined as someone who is currently smoking and had smoked ≥ 100 cigarettes throughout their lifetime. Aerobic exercise was defined as ≥ 1¼ h per week of high intensity physical activity, or ≥ 2½ h per week of moderate intensity physical activity (for those who engaged in both intensities of physical activity, the time engaged in high intensity was weighted as double moderate intensity).

The following health examination covariates were further controlled for: hypertension (systolic blood pressure ≥ 140 mmHg or diastolic blood pressure ≥ 90 mmHg or the use of antihypertensive medication), diabetes (fasting glucose ≥ 126 mg/dL or previous diagnosis by a doctor, or use of antidiabetic medication including insulin injections), hypercholesteremia (fasting total cholesterol ≥ 240 mg/dL or the use of antilipidemic medication)^44^, obesity (body mass index ≥ 25 kg/m^2^)^45^, and anemia (hemogloblin < 12 g/dL for women and < 13 g/dL for men). The definitions for these conditions were based on the guideline of the KNHANES. All these variables were treated dichotomously. Additionally, serum AST, creatinine, and hs-CRP levels were used as continuous covariates.

Factors from the nutritional survey were also controlled for. These included daily energy and daily protein intake, calculated from a 24-h dietary recall of the day before the nutritional survey (midnight to midnight). Calculations were based on the 8th food composition table by the Korean National Rural Resources Development Institute^46^.

### Statistical analyses

Analyses were adjusted for the sampling procedures and weights of the KNHANES complex sample design. For skewed covariates, logarithmic transformations were performed for further analysis. Initially, demographic, clinical, and nutritional characteristics were compared between participants with and without low-grade inflammation. Chi-square tests were performed for categorical variables and *t* tests for continuous variables, with the exception of PHQ-9 scores, which were analyzed using the Kruskal Wallis test. Due to the positive skewness and excess of zeros (37.8%) among participant PHQ-9 scores, we used negative binomial regression to analyze the relationship between serum UA and depressive symptoms. Initially, the statistical significance of the interaction between log-transformed UA and the presence of low-grade inflammation for PHQ-9 data was assessed, controlling for previous covariates. The multivariate analysis was stratified by low-grade inflammation, based on a cutoff value of 1 mg/L of hs-CRP. The multivariate analysis was controlled for age, sex, region, income, education, unemployment, marital status, alcohol use, smoking behavior, aerobic exercise, history of cancer, history of arthritis, hypertension, diabetes, hypercholesteremia, obesity, anemia, serum AST, serum creatinine, serum hs-CRP, daily energy intake, and daily protein intake.

We calculated model fitness by dispersion indices. When calculating these dispersions, weights were standardized to the sample size of each stratified group of low-grade inflammation by dividing individuals’ weights by the mean weight of their stratified group.

Statistical analyses were mainly performed with SSPS Version 23.0 (IBM Corporation, Armonk, NY, USA). STATA Version 15.0 (Stata Corp, College Station, TX, USA) was used for the negative binomial regression, and R Version 3.6.2 (https://www.R-project.org/) was used for the design-based Kruskal Wallis test. Statistical tests were two-sided, and *p* < 0.05 was considered statistically significant, with the exception for interaction analysis, in which *p* < 0.10 was considered statistically significant^47^.

## Data Availability

This study analyzed the data from the KNHANES VII-1 (2016). The data is available to the public after certain procedures (http://knhanes.cdc.go.kr).
